# Tufts PACE Clinical Predictive Model Registry: update 1990 through 2015

**DOI:** 10.1186/s41512-017-0021-2

**Published:** 2017-12-21

**Authors:** Benjamin S. Wessler, Jessica Paulus, Christine M. Lundquist, Muhammad Ajlan, Zuhair Natto, William A. Janes, Nitin Jethmalani, Gowri Raman, Jennifer S. Lutz, David M. Kent

**Affiliations:** 1 Division of Cardiology, Tufts Medical Center, Boston, USA; 2 Predictive Analytics and Comparative Effectiveness (PACE) Center, Institute for Clinical Research and Health Policy Studies (ICRHPS), Tufts Medical Center/Tufts University School of Medicine, 800 Washington Street, Box 63, Boston, MA 02111 USA; 3 King Abdulaziz Cardiac Center, King Abdulaziz Medical City (Riyadh), Ministry of National Guard - Health Affairs, Riyadh, Kingdom of Saudi Arabia; 4 Center for Clinical Evidence Synthesis, ICRHPS, Medical Center/Tufts University School of Medicine, Boston, USA

**Keywords:** Prediction, Cardiovascular disease risk factors, Cerebrovascular disease/stroke, Modeling, Prognostic factor, Clinical predictive model, Coronary artery disease, Risk stratification, Methods

## Abstract

**Background:**

Clinical predictive models (CPMs) estimate the probability of clinical outcomes and hold the potential to improve decision-making and individualize care. The Tufts Predictive Analytics and Comparative Effectiveness (PACE) CPM Registry is a comprehensive database of cardiovascular disease (CVD) CPMs. The Registry was last updated in 2012, and there continues to be substantial growth in the number of available CPMs.

**Methods:**

We updated a systematic review of CPMs for CVD to include articles published from January 1990 to March 2015. CVD includes coronary artery disease (CAD), congestive heart failure (CHF), arrhythmias, stroke, venous thromboembolism (VTE), and peripheral vascular disease (PVD). The updated Registry characterizes CPMs based on population under study, model performance, covariates, and predicted outcomes.

**Results:**

The Registry includes 747 articles presenting 1083 models, including both prognostic (*n* = 1060) and diagnostic (*n* = 23) CPMs representing 183 distinct index condition/outcome pairs. There was a threefold increase in the number of CPMs published between 2005 and 2014, compared to the prior 10-year interval from 1995 to 2004. The majority of CPMs were derived from either North American (*n* = 455, 42%) or European (*n* = 344, 32%) populations. The database contains 265 CPMs predicting outcomes for patients with coronary artery disease, 196 CPMs for population samples at risk for incident CVD, and 158 models for patients with stroke. Approximately two thirds (*n* = 701, 65%) of CPMs report a *c-*statistic, with a median reported *c-*statistic of 0.77 (IQR, 0.05). Of the CPMs reporting validations, only 333 (57%) report some measure of model calibration. Reporting of discrimination but not calibration is improving over time (*p* for trend < 0.0001 and 0.39 respectively).

**Conclusions:**

There is substantial redundancy of CPMs for a wide spectrum of CVD conditions. While the number of CPMs continues to increase, model performance is often inadequately reported and calibration is infrequently assessed. More work is needed to understand the potential impact of this literature.

**Electronic supplementary material:**

The online version of this article (10.1186/s41512-017-0021-2) contains supplementary material, which is available to authorized users.

## Background

Prognosis is an essential task in clinical practice, yet it is a task in which physicians have repeatedly demonstrated poor performance [1]. Clinical prediction models (CPMs), empirically derived from large databases using mathematical models, have been developed to provide objective, patient-specific risk estimates of the probability of important outcomes based on easily ascertained clinical variables. These tools are designed to enable clinicians to “personalize” risk-sensitive medical decisions for individual patients [2]. In cardiovascular disease (CVD) and other fields of medicine, CPMs are now incorporated into numerous clinical practice guidelines [3–5].

Despite the compelling rationale for CPMs, as well as their growth in the literature [6] and increasing incorporation into clinical guidelines, application of these models remains limited and their potential impact on clinical care remains largely unknown. There have been recent collaborative efforts to understand the extent and limitations of the CPM literature, to establish methodological standards [7] and reporting guidelines [8], and to create a community of researchers dedicated to advancing this field, including the creation of a new journal [9]. To contribute to this broad effort to better understand the scope and limitations of the literature, we created the Tufts Predictive Analytics and Comparative Effectiveness (PACE) Clinical Predictive Model (CPM) Registry, which describes published CPMs for patients at risk for and with known CVD. Herein, we report an update to this field synopsis and present an online version of the registry.

The Registry is available at http://pace.tuftsmedicalcenter.org/cpm to aid clinicians and researchers in understanding the state of CPM development across the spectrum of CVD.

## Methods

### Study search and selection

**Fig. 1:**
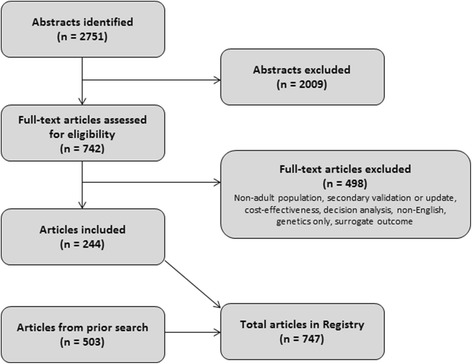
PubMed was searched for relevant articles from 1990 to March 2015

Our search strategy has been previously described [6]. Briefly, we performed a PubMed search for English-language articles containing newly developed CPMs. A CPM was defined as a model that provides a method to calculate or categorize an individuals’ risk for a binary outcome. Here, we updated our search to include articles published through March 31, 2015. We supplemented this search by scanning reference lists to ensure completeness of the database (Fig. 1). CVD includes coronary artery disease (CAD), congestive heart failure (CHF), arrhythmias, stroke, venous thromboembolism (VTE), and peripheral vascular disease (PVD).

For this registry, a CPM is defined as a predictive model that can be used to estimate an individual patient’s absolute risk for a binary outcome. Detailed inclusion and exclusion criteria have been previously reported [6]. The Registry includes articles that describe both prognostic and diagnostic models. Included articles describe CPMs for patients at risk of developing CVD and also for patients with known CVD that predict binary outcomes (e.g., MI, death, or composite endpoints). To be included in the database, articles must include sufficient information about the CPM—typically in the form of a point score, equation, decision tree, nomogram, or online calculator—for readers to be able to generate individual predictions.

### Data extraction

This report focuses on de novo CPMs, defined as newly derived CPMs*.* We extracted CPM information directly into our Microsoft Access 2010 database. Blinded double extractions of key fields were done to ensure consistency of extracted data. Discrepancies were discussed to arrive at a consensus.

We extracted information at the article and model level. All data extraction done after 2015 has been aligned with the recently published Transparent Reporting of a Multivariable Prediction Model for Individual Prognosis or Diagnosis (TRIPOD) statement [8] and Critical Appraisal and Data Extraction for Systematic Reviews of Prediction Modeling Studies (CHARMS) Checklist [10]. Measures of CPM discrimination and calibration were extracted as well as information on the type of validation reported at the time of publication. CPMs were characterized based on index condition/outcome pairs (I/O pairs). CPMs focused on predicting the development of incident CVD were characterized as “population sample” CPMs. Outcomes were categorized as mortality, major adverse cardiac events (MACE), major adverse cardiovascular or cerebrovascular events (MACCE), other composite outcomes, or other clinical events. We created a clinically oriented classification scheme to describe CPM variables based on Medical Subject Headings (MeSH) terms. Where an appropriate MeSH term was not identified, we categorized covariates with an appropriate heading. We present the most common covariates in the Registry (overall) and also across the top 5 index conditions.

We examined secular trends in the reporting of CPM performance. We compared the proportion of models reporting discrimination, some measure of calibration, and inclusion of a web- or computer-based calculator for estimating probabilities across 5-year intervals from 1990 to 2015 using a chi-square test for trend.

## Results

### Overall registry

**Fig. 2:**
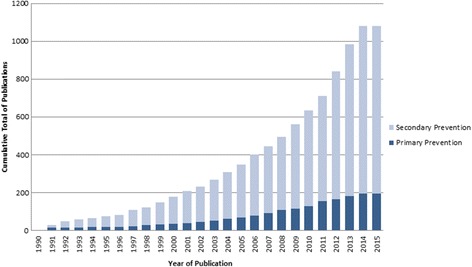
Cumulative growth in published CPM articles included in the Tufts CPM database over time (January 1990–March 2015). Dark blue represents models derived on CVD-free population samples. Light blue represents models derived on patients with specific cardiovascular conditions at baseline

**Fig. 3:**
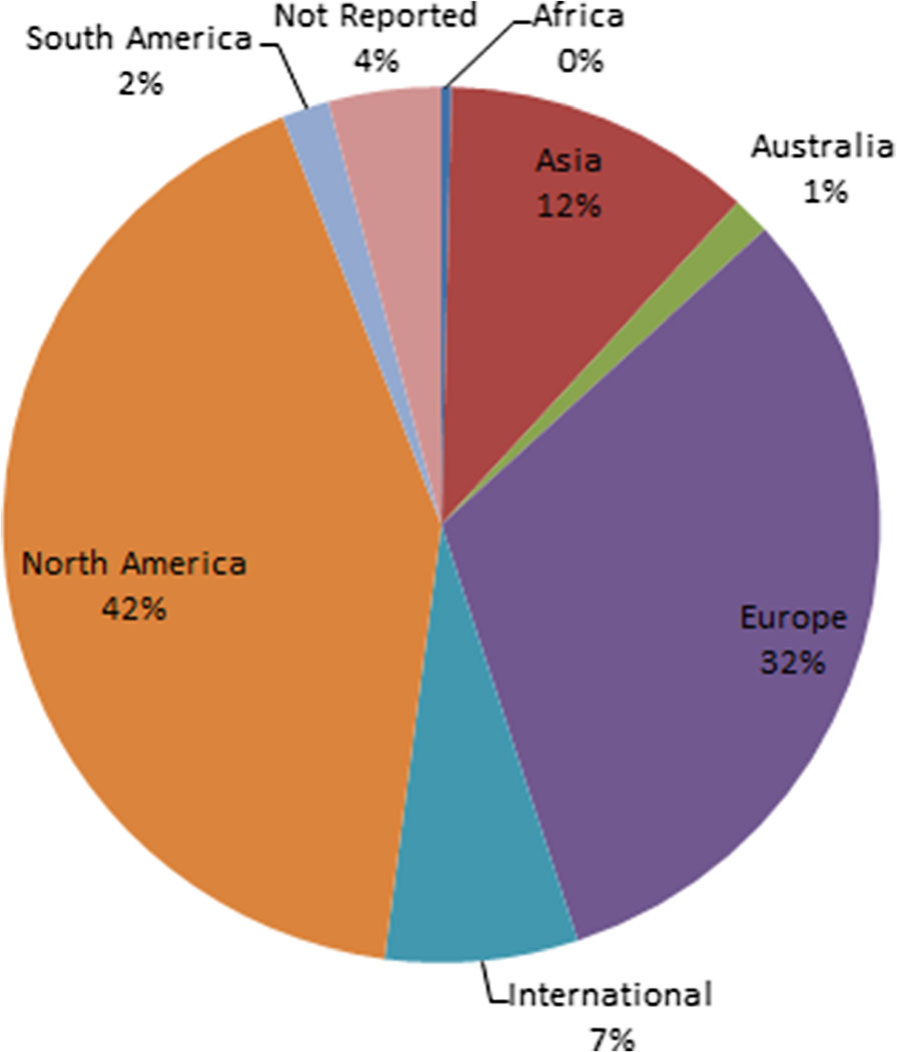
CPMs by derivation cohort geographic region

The Registry includes 747 articles presenting prognostic (*n* = 1060) and diagnostic (*n* = 23) CPMs (Fig. 1). Three hundred seventy-four CPMs were added to the Registry during this update. There was a threefold increase in the number of CPMs published between 2005 and 2014 (the last full decade for which we have data), compared to the prior 10-year interval (1995–2004) (Fig. 2). CPMs were most commonly published in specialty journals (Table 1). *Circulation* published 53 (7.1%) and the *Journal of American College of Cardiology* published 45 (6.0%) of the articles included in the Registry. The majority of CPMs were derived from either North American (*n* = 455, 42%) or European (*n* = 344, 32%) populations (Fig. 3). The most common statistical method used to create CPMs was logistic regression (55%), followed by Cox regression (33%). CPMs were derived from a variety of data sources: 63% derived CPMs from cohort studies, 19% used registry data, and 10% used RCT data. For the top 10 index conditions, CPMs most commonly (51%) predicted mortality over a short time frame (< 3 months). Forty-four percent of these CPMs predicted mortality over a long time frame (> 6 months).

**Table 1:** Journals ranked by number of CPMs published in 1990–2015

Journal	Count	Rank
*Circulation*	53	1
*Journal of the American College of Cardiology*	45	2
*Stroke*	40	3
*American Journal of Cardiology*	36	4
*Annals of Thoracic Surgery*	25	5
*European Heart Journal*	21	6
*American Heart Journal*	21	7
*International Journal of Cardiology*	17	8
*Journal of Vascular Surgery*	16	9
*Journal of the American Medical Association*	15	10
Other	455	
Total	747	

Journals ranked according to number of published CPM articles from 1990 to March 2015. “Other” includes all other journals publishing CPM reports. CPM indicates clinical predictive model

For the 10 most commonly studied I/O pairs, 102 models (18%) did not report the number of events in the derivation cohort (Table 2). For the top ten I/O pair de novo CPMs that reported the number of events, there was a wide range of events per included variable (EPV) (median = 25 [IQR, 12]).

### Clinical focus

**Fig. 4:**
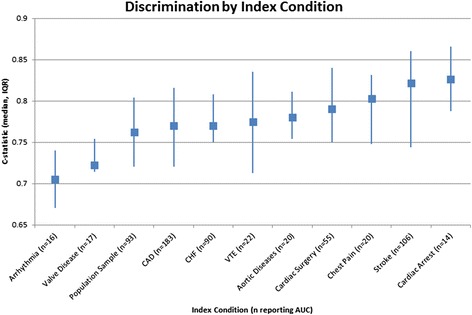
Discrimination of CPMs by index condition. Discrimination is reported as *c*-statistic (median, IQR). CPM, clinical prediction model; CAD, coronary artery disease; CHF, congestive heart failure; VTE, venous thromboembolism

These CPMs represent 24 index conditions and 183 unique I/O pairs. The 10 most frequently studied index conditions and associated summary discriminatory performance are shown in Fig. 4. There are 265 CPMs for patients with known coronary artery disease (CAD), 196 for population samples, and 158 for patients with prior stroke. The diagnostic CPMs included in this database most commonly predict the presence or absence of venous thromboembolic disease (six models) and CAD (four models). Overall, the most commonly predicted outcome was mortality (40%), followed by MACE (9%), stroke (6%), functional outcomes (6%), and MACCE (6%). CPMs predicting mortality were most commonly published for patients with known CAD (126 models), followed by heart failure (100 models) and stroke (51 models). CPMs predicting composite outcomes representing MACE were most frequently developed for population samples (42 models), followed by patients with CAD (27 models) and chest pain (16 models).

### Covariate environment

**Fig. 5:**
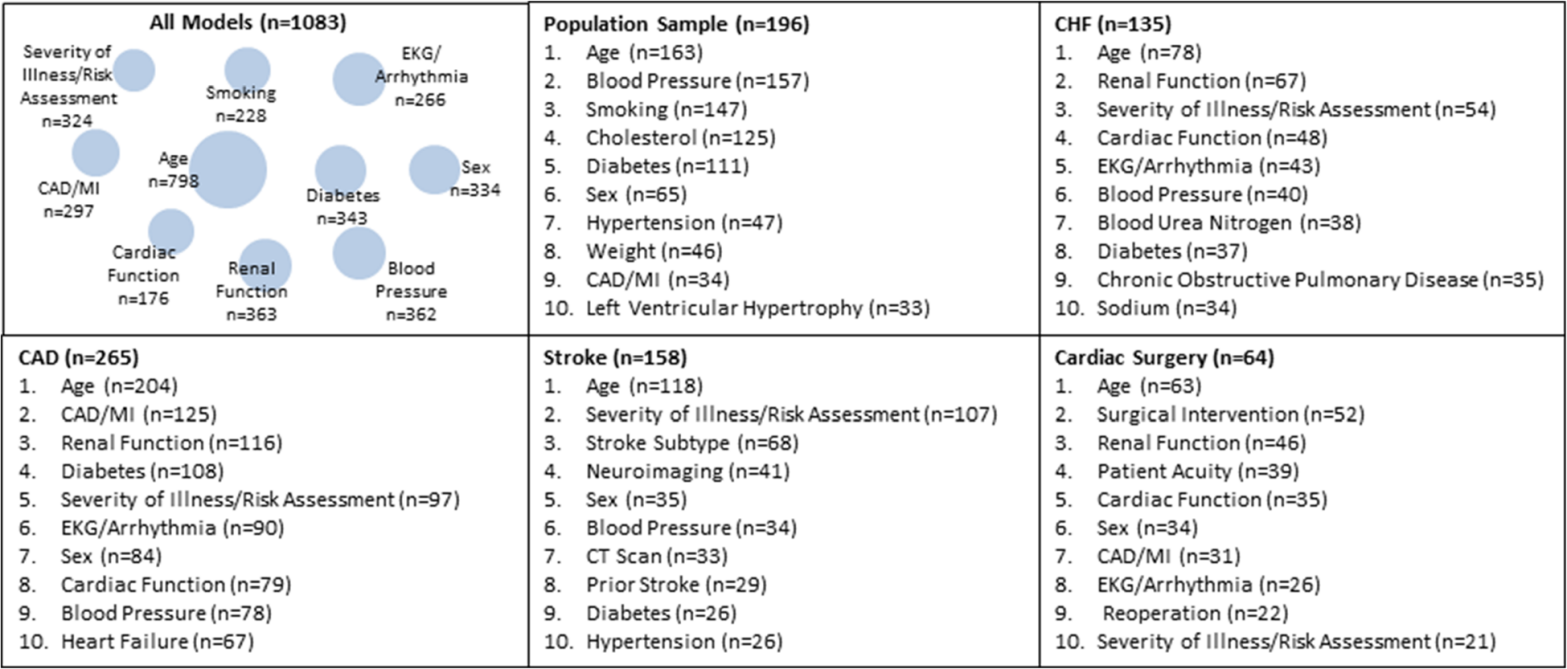
Frequency of covariate categories among all covariates (*n* = 9641) in the Tufts PACE CPM Registry. Top covariates across the top 5 index conditions are also presented

Covariate frequencies are shown in Fig. 5. Across the entire Registry, 798 (74%) of the CPMs include a variable for age. CPMs frequently included covariates representing renal function (34%), blood pressure (33%), CAD/MI (27%), and diabetes (32%). The common covariates are shown for common index conditions. Only 6% of CPMs in this Registry include a covariate for race (counts include interaction terms).

### Model performance

Of the 1083 CPMs in the Registry, 701 (65%) report a *c-*statistic. Discrimination was more frequently reported in logistic regression-based models (455/612, 74%) than in Cox regression models (190/357, 53%). The median reported *c-*statistic was 0.77 (IQR, 0.05). Of the 10 most common index conditions, discrimination was highest for CPMs predicting outcomes following stroke (106/158 reporting, median c-statistic 0.82 [IQR, 0.04]) and cardiac arrest (17/27 reporting, median *c-*statistic 0.83 [IQR, 0.02]) (Fig. 4). Discrimination was lowest for CPMs predicting outcomes for patients with valve disease (17/22 reporting, median *c*-statistic 0.72 [IQR 0.04]). Forty percent (*n* = 433) of CPMs describe an internal validation exercise (including testing on random and non-random subset of the same overall cohort) and 25% (*n* = 274) report validation on a population sample separate from the derivation sample. (This does not include external validations published separately.) Of the CPMs included in this Registry reporting some type of validation (*n* = 577), only 333 (57%) report some measure of model calibration. For the reports presenting an external validation, only 50% report calibration. Of the CPMs reporting calibration that were published after May 2012, only 93 (56%) report a Hosmer-Lemeshow statistic.

### Time trends

The frequency of reporting CPM discrimination as part of the original CPM description increased from 1990 to 2015 (*p* for trend < 0.001). It is also increasingly common to see calculators presented alongside CPMs to enhance clinical use (*p* for trend < 0.01). There is no change over time in the frequency of reporting CPM calibration (*p* for trend = 0.39) (Table 3).

**Table 2:** Index condition/outcome (I/O) pairs of de novo models

I/O pair	Models reporting events	Variables per model	Events per model	Events per variable (EPV)
CAD—mortality	102 (81%)	9 (6–12)	233 (125–709)	35 (15–64)
CHF—mortality	80 (80%)	7 (5–9)	131 (81–253)	24 (13–32)
Population sample—MACE/MACCE	53 (74%)	7 (6–8)	312 (137–686)	36 (20–100)
Stroke—functional outcome	48 (92%)	6 (4–8)	114 (43–310)	16 (9–44)
Stroke—mortality	41 (80%)	5 (4–6)	72 (40–174)	15 (12–42)
CAD—MACE/MACCE	43 (88%)	6 (4–9)	143 (68–254)	21 (13–39)
Cardiac surgery—mortality	31 (97%)	10 (7–13)	171 (95–295)	21 (12–31)
Population sample—mortality	22 (73%)	5 (5–7)	377 (116–1716)	48 (19–343)
Population sample—stroke	18 (69%)	6 (5–8)	227 (112–309)	30 (20–52)
Aortic disease—mortality	23 (92%)	4 (3–7)	43 (26–136)	14 (5–23)

Numbers reported are *n* (%) or median (IQR). Top 10 index condition/outcome (I/O) pairs. We report here variables included in the model (as opposed to candidate variables).

*CAD* coronary artery disease, *CHF* congestive heart failure, *MACE*, major adverse cardiovascular events, *MACCE* major adverse cardiovascular and cerebrovascular events

**Table 3:** Time trends for reporting discrimination and calibration and providing a calculator

Time period	Total models (*n*)	Discrimination		Calibration		Calculator	
		Reporting AUC (%)	*p* for trend	Reporting calibration (%)	*p* for trend	Providing calculator (%)	*p* for trend
1990–1995	75	31	< 0.0001	58	0.39	0	< 0.01
1996–2000	102	49		48		0	
2001–2005	171	61		53		1	
2006–2010	285	72		65		3	
2011–2015	450	71		57		4	

### Website

The Tufts PACE CPM Registry is publically available at http://pace.tuftsmedicalcenter.org/cpm. CPMs are searchable by PubMed ID, index condition, outcome, and Medical Subject Headings (MeSH) terms. Screenshots are shown in the Additional file 1. Extracted information, including sample size, number of events, follow-up duration, and measures of statistical performance, is presented at the model level.

## Discussion

Here, we report updated results from the Tufts PACE CPM Registry and introduce the publically available website, which can serve as a resource to help understand major trends in CPM development. This registry documents continued and accelerating growth of CVD CPMs and an important trend of improved reporting of statistical performance over time. Nevertheless, calibration remains poorly reported (less than 40% of all CPMs and in only 57% of CPMs reporting on validation), despite methodological work emphasizing the critical importance of calibration for decision-making [11].

Perhaps, our most striking finding is the number and continued growth of CPMs in CVD, despite substantial apparent redundancy of models. This growth in the literature likely reflects the increasing ease with which these models can be developed. With the growing volume of and access to research and clinical databases, in addition to the broad availability of software packages, barriers to developing new models are rapidly diminishing. Nevertheless, barriers to clinical translation remain. These barriers are incompletely understood but go well beyond the methodological and statistical issues addressed by prior guidelines [7, 12]. We believe barriers to dissemination relate to whether the rationale for a CPM is strongly linked to a specific decisional context (i.e., if they inform critical decisions), whether its output is informative from a decision analytic perspective [13], whether its output leverages into clinicians’ and patients’ natural decision-making process [14], and how it fits into the highly demanding and sometimes chaotic clinical workflow [15]. While this study did not include any evaluation of the dissemination of CPMs into practice or their influence on clinical decision-making, we suspect that the growth in the number of reported CPMs was not accompanied by a commensurate increase in their use in clinical practice. If CPMs are to deliver on the promise of supporting more individualized evidence-based decisions, better understanding this gap remains an important challenge.

We have created a publically available registry for the research community. Sample screenshots are shown in Additional file 1 and present summary data about each CPM. CPMs are easily searchable by index condition, author, and PubMed ID, and information about model development and performance are readily assessed. Ideally, this Registry will be leveraged when future CPM building is considered to confirm if there is a clinical need for a new CPM and to help identify established predictors of outcomes. The Registry might be consulted by reviewers and editors when evaluating the scientific and clinical merits of new models. The Registry also permits not only the study of CPMs within specific index conditions and outcomes, but also the study of predictor variables of interest across different index conditions [16, 17].

The Prognosis Research Strategy (PROGRESS) group [12, 18] has outlined standards for creating predictive models, and the Transparent Reporting of a Multivariable Model for Individual Prognosis or Diagnosis (TRIPOD) statement [8] has highlighted reporting standards that are increasingly being adopted by journals across a number of disciplines. These important efforts arise from well-documented gaps in reporting [19, 20]. In our systematic review, many more Cox models than logistic models were excluded from the Registry since, in accordance with our original inclusion criteria; many Cox models did not describe the baseline hazard or provide an alternative way to generate individual patient predictions and thus were excluded. Since 2012, 46% of full-text articles that are screened for inclusion are excluded from our Registry because they do report a usable model. In our registry, CPMs remain incompletely reported with relatively little focus on model calibration, despite the important role of good calibration in preventing harmful prediction models [11, 21]. While reporting remains incomplete, we note marked improvements in reporting of discrimination (*c*-statistic) over time. We also document that it is increasingly common to find calculators for bedside use presented alongside CPMs. These reporting observations are notes of optimism for a field that has struggled with reporting consistency.

The final covariates included in the CPMs in this Registry show the importance of common cardiovascular risk factors in predicting outcomes for patients at risk and with known CVD. Renal function is a common covariate across the entire database; however, it is interesting to note that it is less frequently seen in CPMs predicting incident CVD (population sample CPMs) and also CPMs predicting outcomes after stroke. This may be related to the time horizon of prediction or the severity of the index condition. We have previously described that sex is more often included for CPMs predicting incident CVD compared to CPMs for diseased populations [22], an observation that may be partially attributed to the presence of index event bias [23], which may tend to diminish the apparent effects of risk factors among patients selected for the presence of an index event or condition. Ongoing efforts seek to describe these variables at a more granular level and to describe the directionality and effect size of a number of common predictors across various I/O pairs.

Our registry has several limitations. Because there is no MeSH term to identify predictive models, our search strategy may have missed some CPMs that met the inclusion criteria. We are continuing to add CPMs as they are discovered in the course of enhancing the database. Additionally, the registry excluded articles reporting CPMs if they did not provide a means to calculate a prediction (e.g., the authors reported odds ratios but no intercept). Given the level of detailed extraction required to populate the web-based resource and the continued rapid expansion of CPMs, our updated registry now requires further updating. The registry does not focus on CPM validations, so at this time, the performance of these models, outside of the derivation datasets, is generally unknown.

## Conclusion

The Tufts PACE CPM Registry (available at http://pace.tuftsmedicalcenter.org/cpm) is a publically available resource of CVD CPMs. This Registry documents substantial redundancy of CPMs for a wide spectrum of CVD conditions. Model performance is often inadequately reported, though discrimination (but not calibration) reporting appears to be improving over time. More work is needed to understand the potential impact of this literature.

## Additional file

Additional file 1: Website screenshots of Tufts PACE CPM Registry (Accessed 6/4/2017). (DOCX 682 kb)
