# Supplementary figures and images for: Tufts PACE Clinical Predictive Model Registry: update 1990 through 2015
Source: Diagn Progn Res. 2017 Dec 21;1:20. doi: 10.1186/s41512-017-0021-2 (PMC6460840; doi:10.1186/s41512-017-0021-2)

**Additional file 1**

Website screenshots of Tufts PACE CPM Registry (Accessed 6/4/2017)


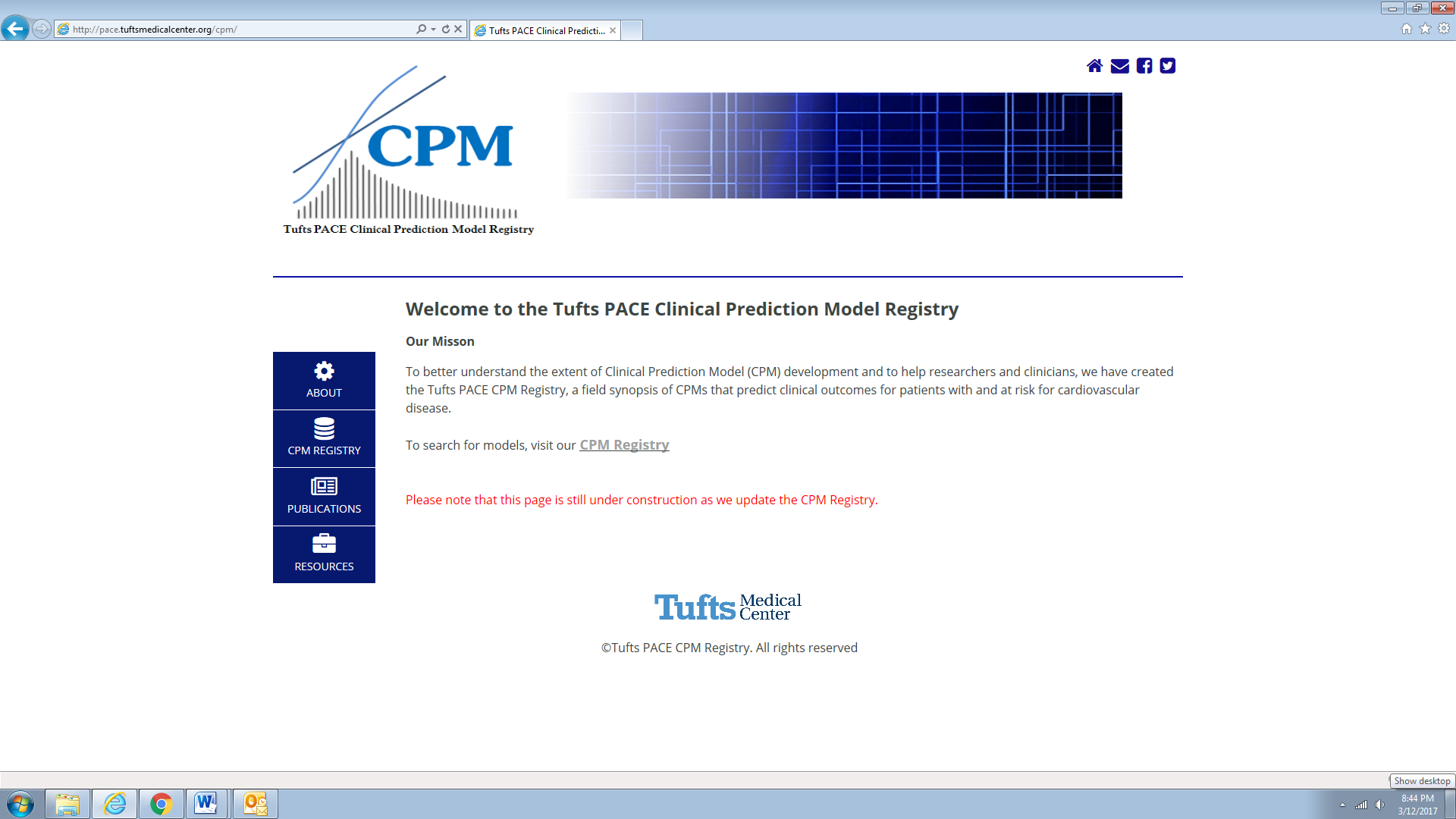


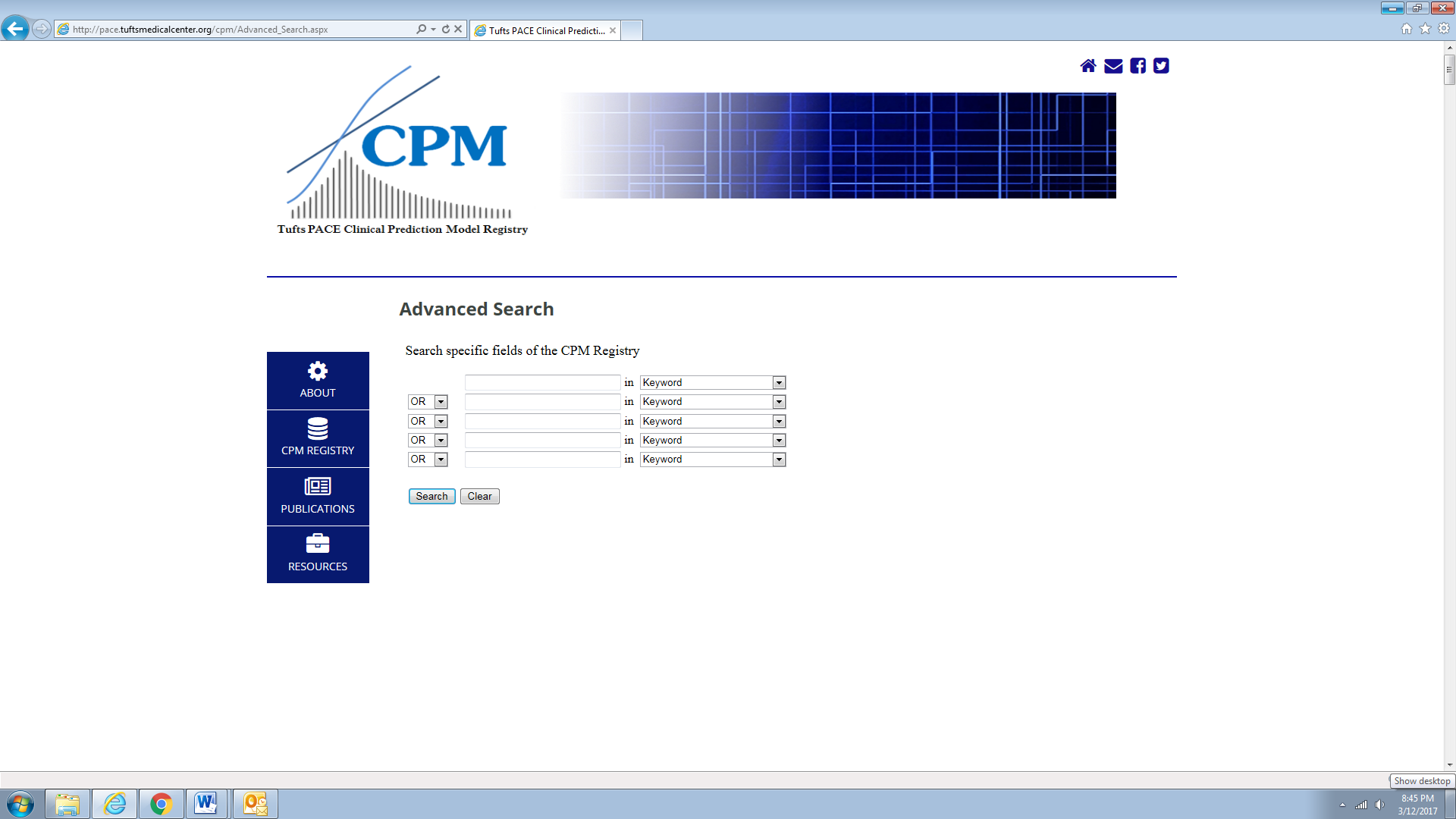


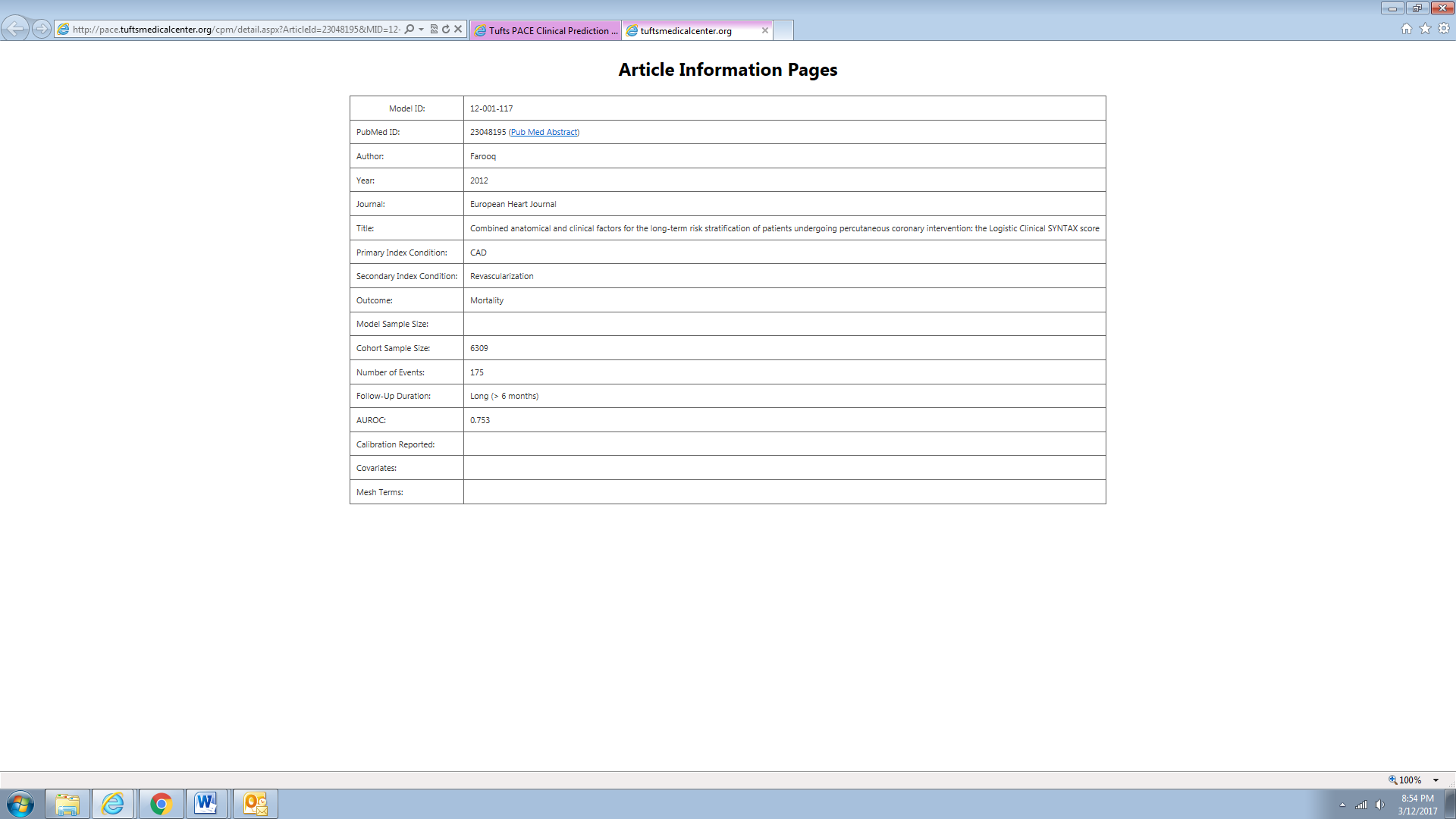

Supplement: Supplementary file 1 — Website screenshots of Tufts PACE CPM Registry (Accessed 6/4/2017). (DOCX 682 kb) [file 41512_2017_21_MOESM1_ESM.docx]
